# Laparoscopic and Robotic Appendectomy: Efficiency and Cost Comparison

**DOI:** 10.26502/jsr.10020519

**Published:** 2026-09-15

**Authors:** Madeleine T Dang, Shaanali Mukadam, Chang Kon Kim, Daniel H Choi, Timothy J Chu, Devendra K Agrawal

**Affiliations:** Department of Translational Research, College of Osteopathic Medicine of the Pacific, Western University of Health Sciences, Pomona, California 91766 USA

**Keywords:** Acute abdomen, Acute appendicitis, Cost effectiveness, Emergency appendectomy, Laparoscopic appendectomy, Minimally invasive surgery, Robotic appendectomy

## Abstract

Acute appendicitis is a common cause of emergency abdominal surgery, most frequently affecting adolescents and young adults. Diagnosis is often complicated by variable clinical presentations, and timely recognition is critical to prevent complications such as perforation, peritonitis, and intra-abdominal abscess formation. Minimally invasive surgery (MIS) has transformed the operative management of appendicitis with laparoscopic appendectomy (LA) established as the standard operative approach due to reduced surgical trauma, faster recovery, lower complications rates, and shorter hospital stay compared to open appendectomy. Robotic appendectomy (RA) has emerged as a newer MIS alternative, offering enhanced visualization, instrument articulation, and ergonomic advantages that may address some technical limitations of conventional laparoscopy. The objective of this review is to examine and summarize current evidence comparing LA and RA in the management of acute appendicitis, including operative time, conversions rates, postoperative recovery, complications, cost, and training considerations. While LA consistently demonstrates favorable outcomes and cost-effectiveness across diverse patient populations, RA may provide incremental benefits in selected cases, particularly those with complex anatomy or increased technical difficulty. However, RA is associated with substantially higher operative and total costs, greater resource utilization, and the need for specialized equipment and trained personnel. By evaluating operative outcomes, patient recovery, and healthcare resource utilization, this review highlights the strengths, limitations, and clinical applicability of both minimally invasive approaches. Current evidence suggests that LA will likely remain the dominant approach for the foreseeable future due to its broad applicability, proven efficacy, and cost effectiveness, whereas RA may continue to grow in niche or complex cases where its technical advantages can be fully leveraged.

## Introduction

Acute appendicitis is one of the most common causes of acute abdominal pain requiring urgent surgical intervention and remains a leading indication for emergency abdominal surgery worldwide. The disease demonstrates a peak incidence among adolescents and young adults, particularly individuals aged 15-19 years, with mortality risk increasing progressively with advancing age [1]. Epidemiology trends also suggest a disproportionate increase in incidence among populations with lower socioeconomic status, highlighting disparities in disease burden and access to surgical care [1,2].

The clinical presentation of acute appendicitis classically involves periumbilical pain migrating to the lower right quadrant with fever, nausea, and leukocytosis; however, presentation may vary depending on patient characteristics such as age and pregnancy status [3,4]. Diagnosis relies on clinical evaluation supported by laboratory findings and imaging modalities, including ultrasound and computed tomography. Timely diagnosis and management are essential to prevent complications such as perforation, peritonitis, and intra-abdominal abscess formation [5]. Management strategies include both operative and nonoperative approaches (Figure 1). Antibiotic therapy may be appropriate in selected patients with uncomplicated disease; however, appendectomy remains the definitive treatment for most cases [3,6]. Operative approaches for appendectomy have evolved substantially, shifting from open procedures toward minimally invasive approaches designed to reduce surgical trauma and improve postoperative recovery [7,8].

Minimally invasive surgery (MIS) has become a central component of modern appendicitis management. These techniques are characterized by smaller incisions, improved visualization of intra-abdominal structures, and reduced physiologic stress compared to traditional open surgery [9,10]. Laparoscopic appendectomy (LA) has emerged as the predominant surgical approach and is widely regarded as the standard surgical treatment for acute appendicitis [8]. LA has gradually gained popularity over the past 10-15 years due to improvements in diagnostic capability and reduced rates of wound-related complications, with no major postoperative complications [11].

Despite its widespread adoption, laparoscopic surgery presents technical challenges related to visualization, instrument maneuverability, and operator learning curves [12,13]. Ongoing technological advancements, including three-dimensional visualization systems, low-impact laparoscopic techniques, and structured training programs, reflect continued efforts to optimize procedural performance and patient outcomes [12,14,15]. Additionally, research has examined patient selection, postoperative recovery, and healthcare access to minimally invasive procedures, emphasizing the importance of equitable implementation and appropriate resource utilization [2].

Robotic appendectomy (RA) represents a more recent advancement in minimally invasive approaches. Robotic platforms incorporate three-dimensional visualization, enhanced instrument articulation, and ergonomic improvements that may address some technical limitations associated with conventional laparoscopy. Robotic surgery has been increasingly adopted in both elective and emergency surgical settings, reflecting broader technological expansion within general surgery [16-18].

The integration of robotic technology into appendectomy has raised important considerations regarding surgical training, operating room logistics, and healthcare resource allocation. Implementation requires specialized equipment, trained personnel, and institutional infrastructure, and its applicability in emergency surgery continues to be investigated [19,20]. Although utilization of RA has increased in recent years, its adoption remains limited compared with laparoscopic approaches, and its clinical value continues to be refined [21].

LA currently represents the standard operative management of acute appendicitis; however, advances in robotic technology and increasing interest in alternative minimally invasive techniques have promoted ongoing investigations into robotic-assisted approaches. Questions remain regarding their relative effectiveness, cost consideration, training requirements, and implications for healthcare systems.

The objective of this review is to evaluate and compare LA and RA with respect to operative considerations, postoperative recovery, complications, healthcare costs and broader clinical implications in the management of acute appendicitis.

## Methods

We conducted a literature search for this narrative review in PubMed, Cochrane, and Embase from November 1, 2025, through January 8, 2026, using the terms “laparoscopic”, “robotic”, “robot-assisted”, “minimally invasive” in conjunction with “appendectomy”, “emergency abdominal”, and “appendix”.

Articles were screened based on title and abstract, followed by full-text review. We included studies primarily published within the last five years that were relevant to the operative management of acute appendicitis, with a focus on comparisons between laparoscopic and robotic appendectomy, as well as associated operative outcomes, postoperative recovery, complications, training considerations, and cost. We excluded studies that focused on nonoperative management of appendicitis as well as those that were not directly relevant to minimally invasive surgical approaches.

### Operative Time in Appendectomy

LA has demonstrated favorable operative times compared with open appendectomy (OA), with outcomes influenced by both surgical technique and patient-specific factors (Figure 2). Ranjan et al. [22] reported that standard LA had the shortest mean operative time (48.7 minutes), followed by single-incision LA (52.3 minutes), while OA required the longest operative time (65.4 minutes). A 2021 prospective, randomized, single-blind, multicenter trial compared two-dimensional and three-dimensional laparoscopy and found that three-dimensional laparoscopy significantly reduced operative time [12].The authors attributed the improved efficiency to enhanced depth perception, which helps overcome a key limitation of conventional laparoscopy [12]. In patients with increased subcutaneous fat thickness, LA was associated with shorter operative times [23]. Similarly, LA has demonstrated favorable operative efficiency in obese populations, further supporting its advantages in patients with larger body habitus [24].

However, not all studies demonstrate clear operative time advantage. Bulut et al. [25] found no statistically significant difference in operative time between LA and OA (53.5 vs. 54.9 minutes), although LA was associated with benefits beyond operative time [25]. Furthermore, in elderly populations, LA may be associated with slightly longer operative times compared to OA [26,27].

RA has been increasingly evaluated as an alternative minimally invasive approach, though its impact on operative time remains variable. Using data from the National Surgical Quality Improvement Program, Becker et al. [28] found that RA had longer operative times than LA; however, it is predicted that operative times will decrease with additional robotic experience. The authors noted that RA accounted for only 0.1% of minimally invasive appendectomies during the study period, emphasizing limited adoption and the influence of early learning curves on operative time [28]. Similarly, a case-based analysis suggests that while RA may require longer operative times, there is potential for improved efficiency as surgeon experience increases [29].

Some comparative studies suggest that the operative time differences between LA and RA may be narrowing. In a 2023 retrospective study, Rifai et al. [30] found that the mean intraoperative time was not significantly different [30]. Similarly, Thomas et al. [31] reported that RA had shorter operative times compared with LA (77.2 vs 86.9 minutes), in addition to several postoperative advantages [31]. However, these findings are not consistent across all settings; a systematic review focusing on RA for complicated appendicitis reported a mean operative time of 128 +/− 32 minutes, which exceeds typical operative times for LA [32].

Overall, LA remains a well-established approach with favorable operative times compared to OA. While RA does not uniformly demonstrate shorter operative times than LA, emerging evidence suggests that with increasing experience and improved technology, RA may offer comparable efficiency alongside advantages in postoperative recovery, conversion rates, and complication profiles. Differences in operative time between LA and RA appear to be strongly influenced by surgeon experience, case complexity, and institutional adoption rather than the surgical platform alone.

### Conversion

Conversion from minimally invasive appendectomy to OA is an important marker of technical difficulty and is associated with increased postoperative morbidity and length of stay [33].

Reported conversion rates from LA to OA range from 2% to 8.7%, with substantial variation based on patient characteristics and institutional experience [32, 34]. Multiple studies have identified consistent clinical, laboratory, and radiologic predictors of conversion in LA. Obesity, prior abdominal surgery, complicated appendicitis, and peritonitis were identified as independent risk factors for conversion following LA [32]. Similarly, Turhan et al. [35] reported that high fever, nausea, elevated Alvarado score, increased neutrophil percentage and C-reactive protein levels, as well as radiologic findings such as periappendiceal fluid and lymphadenopathy were significantly more common in cases that converted to open surgery [35]. In a multivariate analysis, a Modified Clinical Appendicitis Score greater than or equal to 6 was identified as the only independent predictor of conversion, while older age, male sex, diabetes, Black race, and complicated appendicitis were also associated with increased likelihood of conversion [36]. Likewise, Azılı et al. [37] demonstrated that age greater than 65 years, increased American Society of Anesthesiologists (ASA) score, and greater reliance on preoperative CT imaging were significant predictors of conversion from LA to OA [37].

These findings were reinforced in a 2025 systematic review and meta-analysis by Mirdamadi et al. [34], which demonstrated that patients older than 60 years had a two-fold increased risk of conversion, while male sex was associated with a 29% higher risk. Obesity led to a four-fold increase in conversion risk, likely due to excessive intra-abdominal fat and impaired visualization [34]. Higher ASA class (III-IV), prior abdominal surgery, delayed presentation, and elevated inflammatory markers, including white blood cell count, C-reactive protein, neutrophil percentage, and neutrophil to lymphocyte ratio, were also associated with increased conversion risk. Radiologic features, specifically increased appendiceal diameter on preoperative CT imaging, emerged as robust predictors of laparoscopic conversion [34].

Emerging data suggests that RA may be associated with lower conversion rates, particularly in complex cases. In a 2023 retrospective comparison, Rifai et al. [30] reported that among 110 LA, one case required conversion to open surgery, while none of the 81 RA cases required conversion [30]. Similarly, Coco et al. [32] evaluated 371 cases of RA performed for complicated appendicitis reported a conversion rate of 6.5%, which is comparable or lower than rates reported for LA in similar disease severity [32].

Together, these studies highlight that patient factors, disease severity, and preoperative findings strongly influence the likelihood of conversion from LA to OA, while emerging evidence suggests that RA may offer lower conversion rates, particularly in complex cases [30, 32]

### Postoperative Outcomes

#### Surgical Site Infections

LA has consistently demonstrated lower rates of postoperative infectious complications compared with OA. In a large retrospective analysis, Seow et al. [8] found that LA conferred a protective effect against severe postoperative complications, including a reduced incidence of surgical site infections (SSI) [8]. Additionally, a 2022 meta-analysis encompassing 777 studies reported significantly lower overall incidence of adverse events, including infectious complications, with LA compared to OA; this is likely attributable to reduced tissue trauma and avoidance of bowel exteriorization [10]. However, infection risk following LA is not uniform and may be influenced by several patient-specific factors. A 2024 retrospective study identified total, visceral, subcutaneous, and skeletal muscle adiposity as significant predictors of SSI after LA [38]. In patients with increased subcutaneous fat thickness, Teke et al. [23] similarly reported lower SSI rates with LA compared to OA, highlighting the advantages of minimally invasive surgery in obese populations [23]. These benefits have also been demonstrated in patients with a body mass index (BMI) greater than 40kg/m^2^, where LA was associated with significantly lower incidence of wound infection compared with open surgery [24].

Intra-abdominal abscess formation represents another important post-operative infectious outcome. Liu et al. [5] identified appendiceal perforation and prolonged operative time as independent risk factors for postoperative intra-abdominal abscess formation following LA [5]. Despite these risks, the authors emphasized that enhanced visualization and reduced surgical trauma associated with laparoscopy contribute to overall improved postoperative outcomes [5].

Overall, these findings underscore that LA offers significant advantages in reducing postoperative infectious complications, particularly in high risk factors, while patient factors and disease severity continue to influence individual infection risk.

#### Pain, Recovery, Functional Outcomes

Postoperative pain and recovery time are important patient-centered outcomes. Minimally invasive appendectomy has been consistently associated with reduced postoperative pain and faster recovery, and ongoing refinements in surgical technique continues to be investigated to further optimize these outcomes [39]. A 2023 prospective study showed that low-impact LA, utilizing reduced insufflation pressure and micro-laparoscopy, resulted in significantly less abdominal pain 2 hours after procedure than a standard LA [15].

Patient-reported outcome measures further characterize the recovery trajectory after LA. Using the EQ-5D-5L questionnaire, Hougaard et al. [40] found that patient quality of life declines immediately following surgery and is not fully restored until sometime between postoperative days 7 and 30 [40]. These findings challenge the perception that LA is a minor procedure with negligible recovery time and underscores the importance of appropriate patient counseling and consideration of alternative treatment strategies when appropriate (Figure 3).

In pediatric and small-bodied patients, surgical approach and instrument selection can further optimize outcomes. Research from pediatric robotic surgery suggests that smaller instruments (e.g., 5mm instead of 8mm) mitigates the challenges posed by limited working space without increasing complications, operative time, or assisted port utilization [41]. Smaller instruments and minimized wound tension reduce incision size, which can improve postoperative comfort, cosmetic outcomes, and recovery; emphasizing that these technical considerations should be a deliberate focus, rather than a byproduct of robotic technology [41].

Functional recovery and patient satisfaction are closely linked to incision-related factors, including cosmetic outcomes, comfort, and patient preference. Kim et al. [42] demonstrated superior body image perception and cosmetic satisfaction at six weeks following single-port LA compared with multiport approaches, although overall scar satisfaction was similar between groups [42]. Single-port procedures in this study were also associated with significantly decreased analgesic use on the day of surgery compared to standard LA [42]. Furthermore, qualitative patient-reported data further emphasizes the importance of incision placement and perioperative education. Xu et al. [43] found that patients preferred avoiding subxiphoid and umbilical incisions due to discomfort during movement, and 87.5% favored transverse incisions over vertical incisions for specimen extraction. Notably, 75% of patients reported unmet preoperative educational needs related to incision care and postoperative expectations, emphasizing an opportunity to improve patient-centered perioperative communication [43].

#### Length of Stay/Time to Discharge

Reduced length of hospital stay is one of the most consistent benefits of minimally invasive appendectomy [39]. Li et al. [44] found that factors influencing discharge time following LA include: patient age, postoperative leukocyte count, monocyte count, lymphocyte count, neutrophil count, appendix diameter, and systemic inflammatory response index [44]. Multiple studies demonstrate that LA is associated with significantly shorter postoperative hospitalization compared with OA. In a 2025 retrospective cohort study, the mean length of stay was reported to be 1.92 days for LA compared to 2.82 days for OA [25]. Similar findings were observed by Teke et al. [23] and Seow et al. [8], both reported shorter hospital stays and an earlier return to normal activity with LA [8, 23].

Same-day discharge following LA has emerged as a safe and effective strategy in selected patients. A 2022 randomized controlled trial demonstrated that 99% of patients undergoing LA could be safely managed as outpatients, with shorter lengths of stay and a lower complication rate compared to patients who were in the hospitalized group [45]. These findings were supported by Qurashi et al. [46], who found no increase in complications, readmissions, or unplanned reoperations among adults discharged on the day of surgery compared with those discharged the following day [46]. Although Kashyap et al. [47] did not exclusively study pediatric patients, the cohort was predominantly pediatric, with mean ages of 11.5 and 11.9 years in the overnight observation and same-day discharge groups, respectively; no differences in 30-day postoperative outcomes were observed between the two groups [47].

When comparing LA and RA, several studies suggest that RA may further reduce postoperative length of stay. RA has been associated with a significant reduction in postoperative length of stay compared to LA with an adjusted odds ratio of −0.27 [48]. Likewise, Rifai et al. [30] reported a significantly shorter time to discharge with RA compared with LA (28.3 hours vs 38.3 hours), despite similar intraoperative times. However, large healthcare system analyses suggest that LA continues to demonstrate the shortest overall hospital stay (3.7 days) compared to OA or RA (11.1 days and 5.0 days, respectively) [49].

### Special Population

#### Pregnancy

Appendicitis during pregnancy presents unique diagnostic and operative challenges due to physiologic changes, altered anatomy, and concerns regarding fetal safety. Historically, pregnancy was considered a relative contraindication to laparoscopic surgery due to concerns that elevated intra-abdominal pressure during pneumoperitoneum could reduce venous return and cardiac output, potentially compromising fetal blood flow and increasing the risk of fetal acidosis [50]. However, more recent evidence supports the safety of LA during pregnancy. When investigating maternal and fetal outcomes in LA and OA, Özdemir et al. [51] found no preterm labor was observed in patients undergoing LA; LA was associated with similar obstetric outcomes and complications compared to OA [51]. A 2022 systematic review of LA in pregnancy compared to open surgery found no significant differences in treatment of appendicitis between LA and OA regarding complication rate, clinical characteristics, gestational age at delivery, hospital stay, or birth weight [52]. Similarly, a case-matched analysis comparing pregnant and nonpregnant patients undergoing LA found that the primary difference was a slightly increased length of stay in pregnant patients, while obstetric outcomes were favorable in the majority of cases [53]. Studies have also demonstrated that LA is associated with faster postoperative recovery and lower wound infection rates compared with open surgery in pregnant patients [50].

Additional research suggests that LA can be safely performed throughout pregnancy. Chwat et al. [54] reported that no gestational age limit has been clearly established for laparoscopic appendectomy, although increasing uterine size may present technical challenges later in pregnancy. Importantly, the authors found no significant differences in surgical time, conversion rates or postoperative complications across trimesters [54].

Despite these reassuring findings, appendicitis during pregnancy continues to present important clinical considerations. Surgical intervention carries risks including preterm delivery and fetal loss; however, delaying surgery increases the risk of appendiceal perforation and associated maternal and fetal complications [55]. Therefore, maintaining a low threshold for surgical intervention remains important to minimize the risk of disease progression and optimize outcomes for both mother and fetus.

#### Pediatric Populations

Minimally invasive appendectomy has also become widely adopted in pediatric surgery. Comparative studies have demonstrated that LA is associated with fewer postoperative complications and shorter hospital stays compared with OA in children [56]. However, conversion from laparoscopic to open surgery in pediatric populations may be associated with increased complications [56].

Patient understanding and expectations may also influence perioperative experience in pediatric populations. A questionnaire-based study of patients aged 10–16 years found that only 17% of children were aware of LA prior to hospital admission, although nearly all reported feeling well informed after surgical explanations were provided [57]. Younger children demonstrated limited understanding of the surgical technique but were primarily concerned about incision size, scarring and the need for suture removal, highlighting the importance of age-appropriate patient education and communication [57].

#### Training

LA and RA have become key procedures for surgical trainees, providing a platform to evaluate resident learning curves, operative proficiency, and patient safety. LA is often one of the first procedures performed by trainees, and studies consistently demonstrate that supervised trainees can perform the procedure safely without compromising postoperative outcomes [58]. Operative efficiency improves with experience, particularly in less complex cases, although a plateau in operative time may occur across training years; importantly, trainee participation does not substantially increase operative time or length of hospital stay, with supervised residents performing comparably to attending surgeons [58].

Structured training programs for residents with limited laparoscopic experiences, such as a 5-day course using box trainers or wet labs, have been explored to enhance technical exposure and procedural confidence. While these programs improve resident familiarity and comfort with LA, they do not appear to significantly alter postoperative outcomes for patients [14]. Mastery of LA generally requires completion of 11-35 cases, with senior residents achieving competence earlier than junior residents due to cumulative surgical experience [13]. Procedural volume remains a critical factor in achieving proficiency; Nikolovski et al. [59] reports that performing at least 20 LA cases is important for meeting competency benchmarks in gastrointestinal surgery [59].

While LA provides the foundation for MIS training, RA introduces additional technical complexity and training considerations. There is currently no universally validated robotic surgery curriculum, and adoption can be limited by cost and rotation scheduling [60]. Simulation-based education, including dual-console training and robotic surgery simulators, has been shown to improve resident familiarity with console operation, bedside assistance, and intraoperative decision-making without increasing operative time or patient morbidity [60]. Systematic reviews indicate that the learning curve in robotic surgery is influenced by prior surgical experience, with more experienced surgeons requiring fewer procedures to reach proficiency [61].

Newer robotic platforms, such as the CMR Versius system, have been shown to be safe and feasible for appendectomy and other abdominal procedures, with operative times comparable to established robotic systems [62, 63]. These platforms may expand access to robotic training and facilitate integration of robotic techniques into general surgical education.

Early exposure, regular practice in simulations labs, and structured curricula are recommended to prevent skill atrophy and optimize resident competency as robotic surgery becomes increasingly integrated into general surgical training [60]. Overall, training for emergency MIS is poorly standardized and poorly investigated [64]. Collectively, these findings underscore the need for a structured, competency-based curriculum that integrates both laparoscopic and robotic techniques, particularly in emergency settings, to ensure consistent surgical proficiency and safe patient outcomes.

#### Costs

Cost remains a major consideration when comparing minimally invasive approaches to appendectomy, particularly given the relatively low complexity and reimbursement associated with the procedures. Current evidence suggests that LA offers greater cost efficiency than RA, which is associated with substantially higher operative and total costs, while clinical outcomes appear broadly comparable between the two approaches [17].

Although LA is associated with higher operative costs compared with open appendectomy, it remains economically justified due to improved clinical outcomes and patient-centered benefits [65]. Furthermore, a meta-analysis by Zhang et al. [10], which included 777 studies evaluating the clinical efficacy of LA in acute appendicitis, demonstrated that despite higher operative costs, shorter hospitalizations resulted in comparable overall expenditure compared to OA [10]. Building on these findings, LA demonstrates favorable cost-effectiveness, particularly when surgical workflows are optimized. Kim et al. [66] evaluated efforts to reduce the supply costs through standardization of laparoscopic equipment and found that implementation of standardized equipment sets reduced total intraoperative costs by 25%. These findings highlight opportunities to further improve the economic value of LA through institutional standardization and efficiency measures [66] (Figure 4).

In contrast, RA is consistently associated with higher costs and increased resource utilization. Quilici et al. [67] reported that RA had a higher average direct cost per case ($7,894) and average total cost per case ($13,210) compared with LA ($3,081 and $7,709, respectively). Based on their value-based analysis, the authors concluded that routine use of RA is not supported [67]. Similarly, Grimsley et al. [20] found that adjusted surgical costs were significantly higher for RA ($6,915 ± 2,051) versus LA ($4719 ± 1,935) [20]. Additional contributors to the increased cost of RA include the high upfront capital investment required for robotic systems, ongoing maintenance expenses, and the need for a fully trained support staff available 24 hours per day to maintain an effective robotic emergency surgery program [20]. Instrument-related costs also represent a significant and often underestimated contributor. Robotic instruments are associated with high per-case costs due to limited reuse cycles, as well as costs related to premature instrument failure, replacement and reprocessing requirements [68].

Overall, while both LA and RA provide minimally invasive alternatives to open surgery, LA remains the more cost-effective option. RA is associated with higher operative and total costs, greater resource utilization, and uncertain incremental benefits, while LA provides favorable clinical outcomes with lower costs and opportunities for further cost reduction through procedural standardization [66,67].

## Conclusion

MIS has transformed the management of acute appendicitis, with LA firmly established as the standard operative approach. Across operative efficiency, postoperative recovery, complication profiles, and cost considerations, LA consistently demonstrate that minimally invasive techniques provide meaningful advantages over open surgery, including shorter hospital stays, reduced SSI, earlier return of bowel function, decreased postoperative pain, and favorable expenditures when hospitalization costs are considered [2, 9, 10].

Despite these benefits, access to MIS remains inequitable; elderly patients, those with greater comorbidities, individuals lacking private insurance, rural residents, and patients with lower income are significantly less likely to receive MIS [2]. Rural residence, low income and lack of private insurance status appear strongly interdependent, suggesting that socioeconomic barriers play a substantial role in limiting access. Similarly, patients with disabilities have been shown to undergo MIS at lower rates compared with patients without disabilities across multiple general surgical procedures, including appendectomy [9]. These findings are particularly concerning given that the populations least likely to receive MIS are often those who may benefit most from its reduced physiologic stress, faster mobilization and shorter hospitalization. Furthermore, laparoscopy remains underutilized in emergencies worldwide, representing less than 20% of major emergency operations in some settings, underscoring persistent gaps between evidence and implementation [69].

Robotic-assisted appendectomy represents an evolving extension of MIS. While emerging data suggest that robotic approaches may offer comparable operative times, low conversion rates, and potentially shorter lengths of stay in selected settings, these benefits must be weighed against substantially higher direct and total costs, significant capital investment, and around-the clock trained personnel [20, 67]. Currently, the available evidence does not demonstrate clear superiority of RA over LA sufficiently to justify routine use, particularly for high-volume, relatively low-reimbursement emergence procedures.

Training and system-level factors will continue to influence the future trajectory of minimally invasive appendectomy. Procedural exposure during residency varies across geographic regions, though not strictly determined by national income classifications, suggesting that institutional priorities and educational structures may be as influential as national economic status [70]. Targeted training initiatives, particularly in emergency laparoscopy surgery may expand safe MIS adoption and improve access [69]. Looking forward, innovations such as telesurgery for RA, particularly in technically demanding populations such as pediatric patients, introduce possibilities for expanding surgical expertise across geographic boundaries. Nevertheless, concerns regarding network latency, surgeon adaption and infrastructure reliability must be addressed before widespread implementation [71].

Overall, LA remains the most evidence-supported, cost-effective and broadly applicable minimally invasive technique for acute appendicitis. RA demonstrates technical feasibility and potential incremental benefits in selected contexts, but its widespread adoption requires further high-quality comparative studies, cost-effectiveness analyses and evaluation of long-term outcomes.

This review has several limitations. First, as a narrative review, it is subject to potential selection and publication bias despite a structured literature search. Second, there is a limited number of high-quality studies directly comparing RA and LA, which constrains definitive conclusions regarding their comparative effectiveness. Additionally, there is limited data specifically addressing emergency minimally invasive appendectomy, despite its clinical importance in acute appendicitis [72].

Future efforts should prioritize equitable access to MIS, structured training in emergency settings, and thoughtful integration of advancing technologies to ensure that innovation translates into meaningful improvements in patient-centered care. At the same time, the development of standardized, competency-based training in emergency laparoscopy will be essential to ensure consistent surgical proficiency and safe addition of MIS across institutions.

Ultimately, as surgical techniques and technologies continue to evolve, a balanced focus on evidence-based practice, accessibility and education will be critical to optimizing outcomes in the management of acute appendicitis.

## Figures and Tables

**Figure 1:**
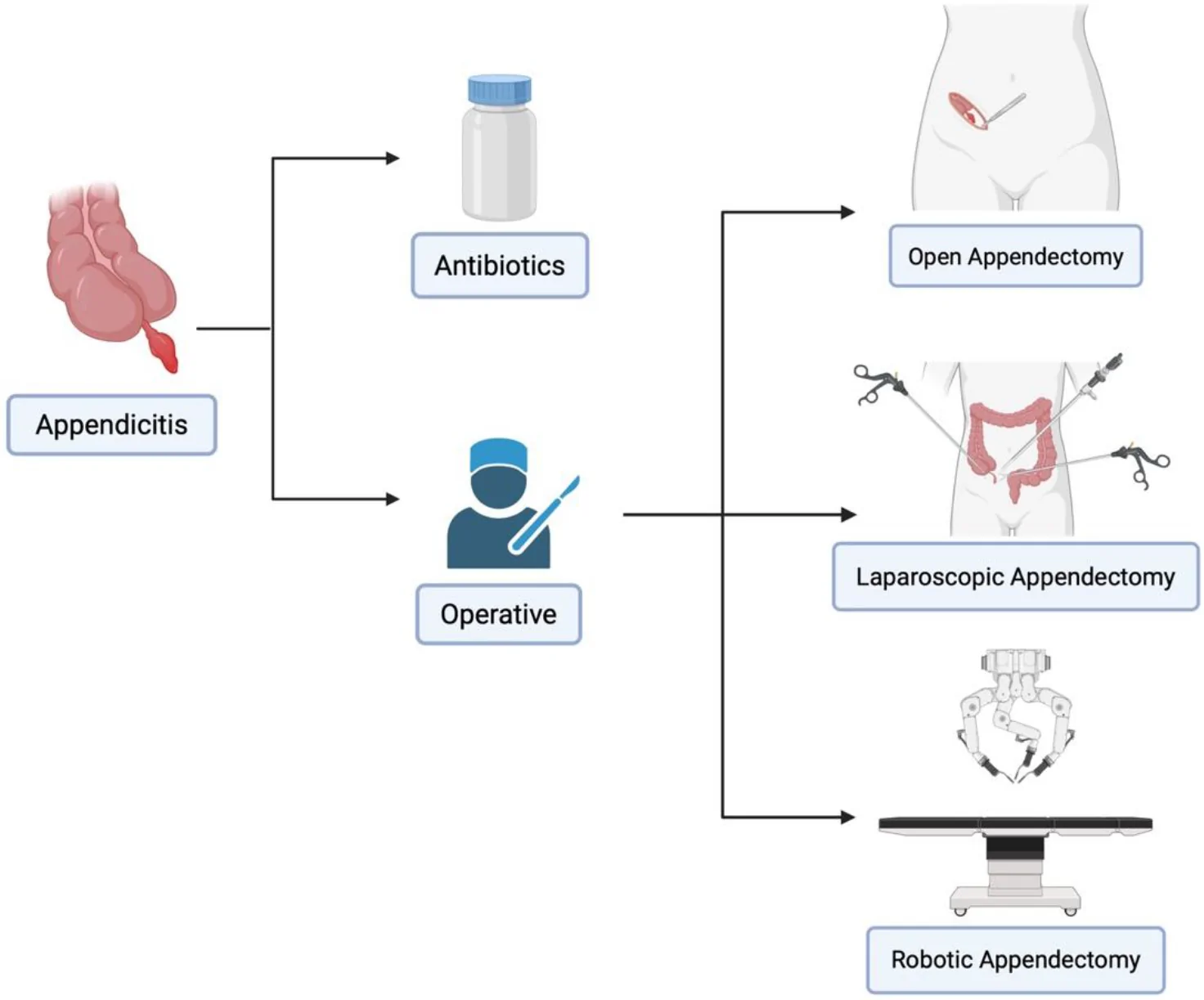
Clinical management pathway for acute appendicitis. Management options include nonoperative antibiotic therapy in selected cases and operative intervention, with open appendectomy, laparoscopic appendectomy, and robotic appendectomy as possible options.

**Figure 2:**
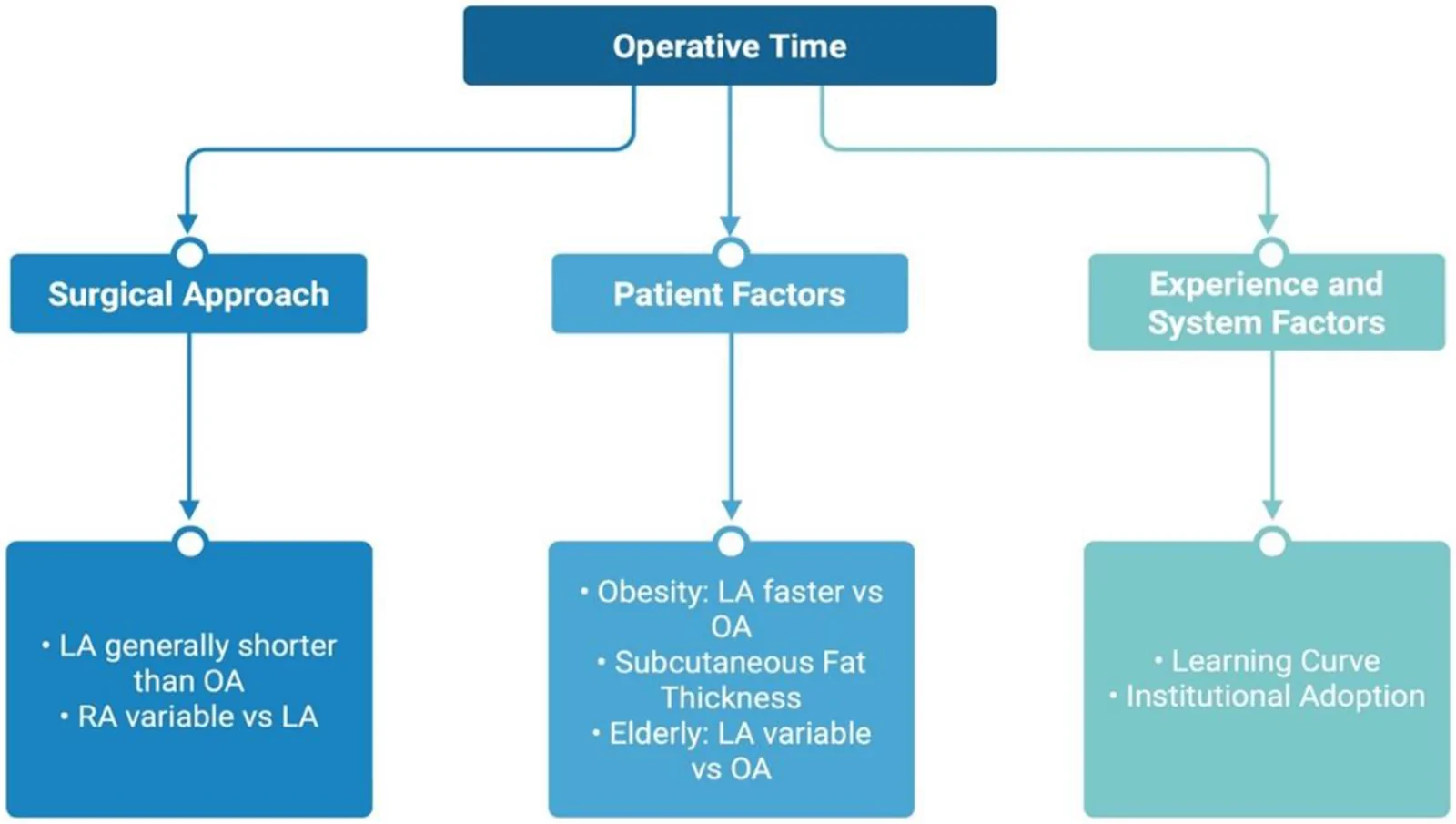
Factors influencing operative time in appendectomy. Conceptual diagram illustrating the multifactorial determinants of operative time in appendectomy. Operative duration is influenced by surgical approach, patient-specific factors, and experience-related variables.

**Figure 3:**
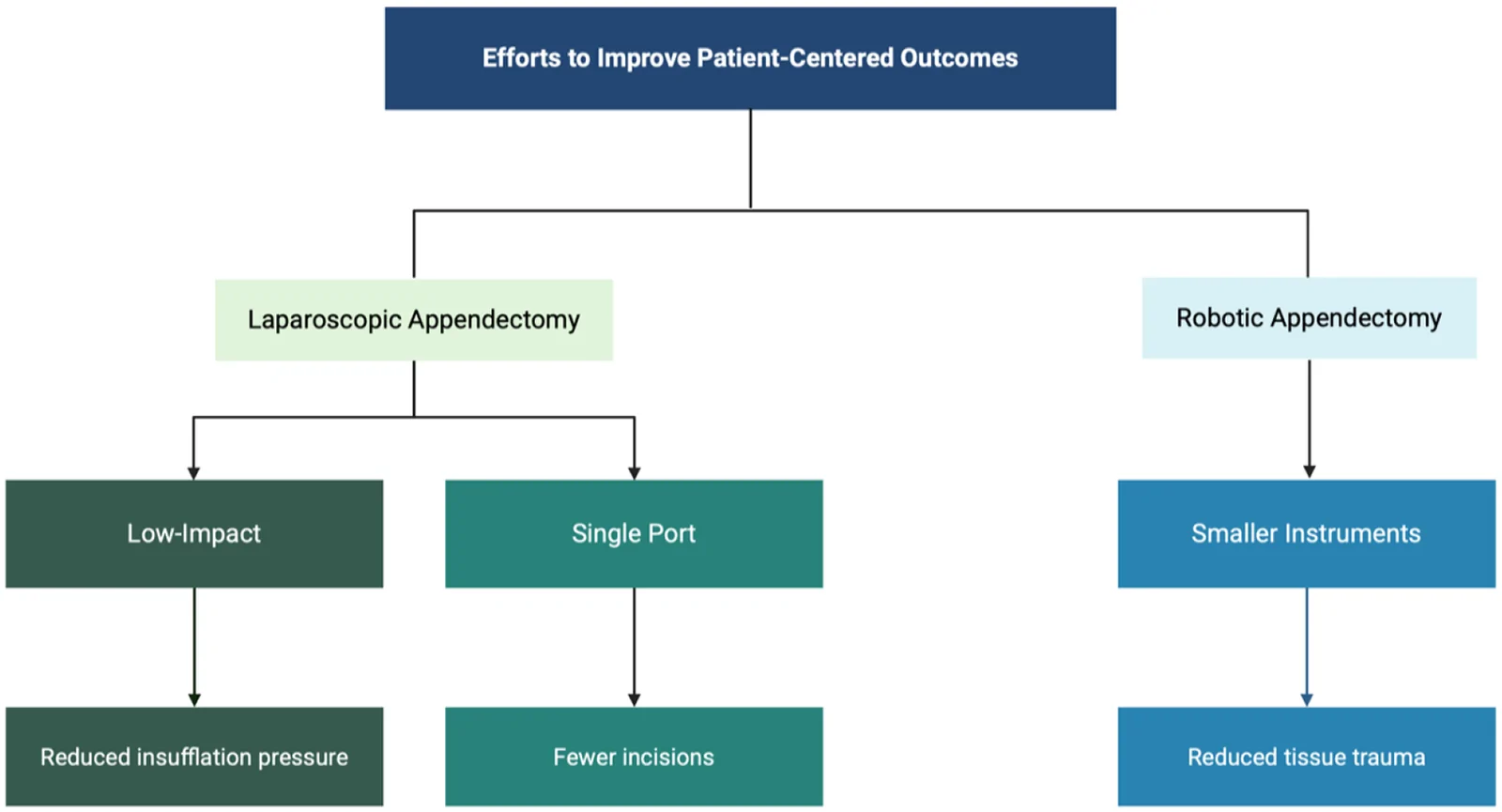
Strategies to improve patient-centered outcomes in minimally invasive appendectomy. Laparoscopic innovations include low-impact techniques and single-port techniques. Robotic approaches emphasize the use of smaller, more precise instruments, which can reduce tissue trauma and enhance surgical precision.

**Figure 4:**
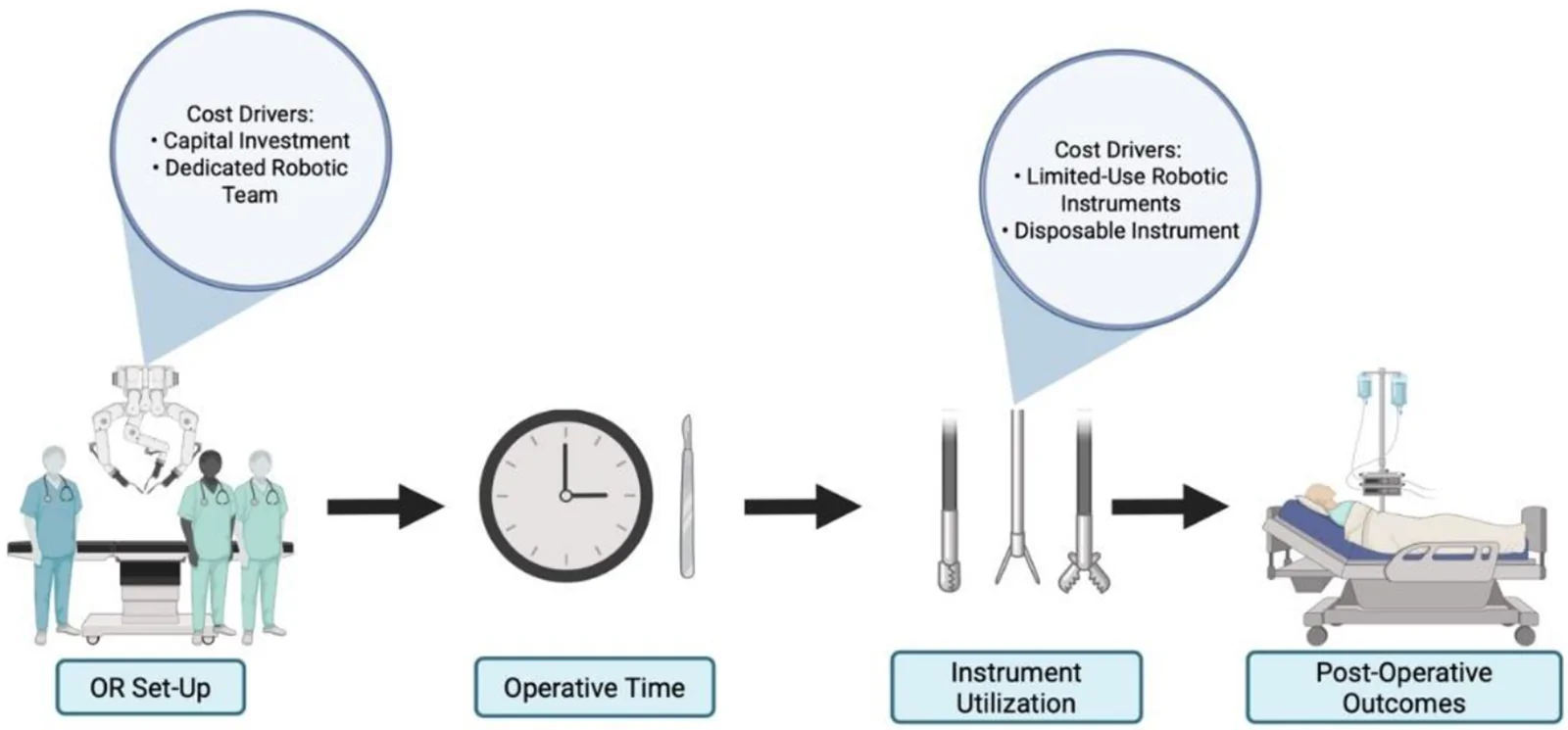
Workflow of robotic appendectomy and associated cost drivers. Major cost drivers include robotic system acquisition and maintenance, specialized instrumentation and the need for trained personnel.

## Key Points

- Laparoscopic appendectomy remains the standard of care for acute appendicitis, offering consistently better outcomes than open surgery, including reduced pain, fewer complications, and shorter hospital stays. Its broad applicability and strong evidence base support its continued dominance in clinical practice.
- Robotic appendectomy provides technical advantages such as enhanced visualization, improved instrument articulation, and better ergonomics for surgeons. However, these benefits have not consistently translated into superior clinical outcomes compared to LA.
- Operative time differences between LA and RA are variable and largely depend on surgeon experience and case complexity rather than the surgical platform itself. While RA may initially require longer operative times, efficiency tends to improve with increased experience and technological advancements.
- RA may offer lower conversion rates to open surgery, particularly in complex or technically challenging cases. In contrast, LA has low conversion rates overall but is more influenced by patient-specific factors such as obesity, prior surgery, and disease severity.
- Both LA and RA improve postoperative recovery compared to open surgery, with reduced pain, shorter hospital stays, and faster return to normal activities. Advances in minimally invasive techniques continue to further optimize patient-centered outcomes and recovery trajectories.
- Cost remains the most significant limitation of RA, as it is associated with substantially higher operative and total expenses due to equipment, maintenance, and staffing requirements. In comparison, LA is more cost-effective while achieving similar clinical outcomes.
- LA is expected to remain the dominant surgical approach due to its accessibility, cost-effectiveness, and proven efficacy. RA will likely continue to develop a more selective role in complex cases where its technical advantages can be fully utilized.
